# Integrated metabolomics and microbiome analyses reveal nutritional differences between yellow and white maize (*Zea mays* L.) kernels

**DOI:** 10.1016/j.fochx.2026.104596

**Published:** 2026-10-07

**Authors:** Qingshan Li, Yu Zu, Hongda Liao, Haidong Huang, Kexin Lu, Lan Lan, XiQin Wei, Shangdong Yang

**Affiliations:** a Guangxi Key Laboratory of Agro-Environment and Agro-Products Safety, National Demonstration Center for Experimental Plant Science Education, Guangxi Agricultural College, Guangxi University, Nanning 530004, PR China; b Economic Crop Station, Bureau of Agriculture and Rural Affairs of Hechi County, Guangxi 533900, PR China

**Keywords:** *Zea mays* L., Endophytic microbiome, Carotenoids, Untargeted metabolomics, Plant-microbe interactions

## Abstract

Maize (*Zea mays* L.) is a globally significant cereal crop that exhibits wide phenotypic and nutritional diversity. Yellow maize is highly valued for its accumulation of health-promoting carotenoids, whereas the functional profile of white maize is characterized by distinct classes of secondary metabolites. However, the complex interplay among host genetics, the kernel endophytic microbiome, and the associated metabolic profiles remains poorly understood. In this study, we employed an integrated approach combining targeted carotenoid quantification, 16S rRNA and ITS amplicon sequencing, and untargeted metabolomics to comparatively analyze the internal microenvironments of yellow and white maize kernels. Phenotypic analysis confirmed that yellow maize kernels accumulated substantially higher concentrations of lutein (up to 5.45 μg/g) and zeaxanthin (up to 15.30 μg/g) relative to the trace amounts detected in white maize. Microbiome profiling revealed that host genotype profoundly shaped endophytic community assembly: although bacterial alpha diversity was comparable between the two groups, yellow maize harbored significantly higher fungal alpha diversity. Taxonomic analysis identified distinct microbial biomarkers, with *Fusarium* being identified as a fungal biomarker enriched in yellow maize, whereas *Aspergillus* was enriched in white maize and *Pseudomonas* dominated its bacterial community. Furthermore, untargeted metabolomics identified 313 differentially accumulated metabolites, predominantly comprising lipids, organic acids, and phenylpropanoids. KEGG enrichment analysis demonstrated that pathways related to phenylalanine, tyrosine, and tryptophan biosynthesis, plant hormone signal transduction, and ABC transporters were significantly enriched, indicating divergent metabolic profiles between the two cultivar groups. Collectively, these findings reveal consistent associations between host secondary metabolism and selectively enriched endophytic microbiomes, providing novel metabolic and microbial targets for future precision breeding and nutritional biofortification in maize.

## Highlights

• Yellow maize kernels accumulated up to 5.45 μg/g lutein and 15.30 μg/g zeaxanthin.
• White maize contained only trace carotenoids but distinct phenolic-rich profiles.
• *Fusarium* and *Aspergillus* were fungal biomarkers of yellow and white maize.
• 313 differential metabolites were linked to carotenoid and phenylpropanoid pathways.

## 1 Introduction

Kernel colour and nutritional composition are critical quality attributes of maize (*Zea mays* L.) that significantly affect both dietary health outcomes and commercial applications. Yellow and white maize, both members of the grass family Poaceae (Ranum et al., 2014), represent contrasting nutritional profiles. Yellow maize is naturally enriched with provitamin A carotenoids, lutein, and zeaxanthin (Muzhingi et al., 2008; Wurtzel et al., 2012), making it an ideal candidate for biofortification initiatives. Carotenoid content is a major determinant of grain quality and dietary value in yellow maize varieties (Nuss & Tanumihardjo, 2010). In contrast, white maize, the preferred staple in many regions worldwide, is valued for its culinary properties and distinctive phenolic profiles, which derive largely from specific phenylpropanoid derivatives (Awika, 2011; Gwirtz & Garcia-Casal, 2014).

The nutritional superiority of yellow maize stems primarily from its carotenoid composition, particularly β-carotene, lutein, and zeaxanthin (Nuss & Tanumihardjo, 2010; Pixley et al., 2013). Variation in total carotenoid accumulation and biofortification efficacy among accessions largely reflects differential carotenogenesis during endosperm maturation (Diepenbrock et al., 2021). During late kernel development, carotenoid levels increase sharply through the coordinated action of enzymes such as phytoene synthase, lycopene ε-cyclase, and *β*-carotene hydroxylase, which collectively regulate isoprenoid metabolism and partitioning in seed sink tissues (Bai et al., 2009; Yan et al., 2010). Multiple quantitative trait loci (QTLs) associated with provitamin A and individual carotenoids have been mapped in diverse maize populations (Owens et al., 2014; Suwarno et al., 2014), and recent transcriptomic studies have identified candidate transcription factors linking gene expression to carotenoid accumulation (Harjes et al., 2008). Furthermore, environmental and hormonal signals modulate the expression of these metabolic genes, contributing to the phenotypic diversity of carotenoid profiles in cultivated yellow maize (Ortiz et al., 2016).

White maize exhibits a distinct nutritional profile characterized by structurally diverse phenolic compounds and complex carbohydrates, including ferulic acid, anthocyanins, and resistant starches (Kgoale et al., 2026). Numerous bioactive secondary metabolites have been identified in white maize, with several key compounds directly linked to its antioxidant capacity and processing quality (Žilić et al., 2012). These phytochemicals are synthesized through specialized phenylpropanoid biosynthetic pathways and subsequently undergo extensive enzymatic and non-enzymatic modifications, generating a diverse array of functional constituents (Pusztahelyi et al., 2015). Metabolomic comparisons among cultivars suggest that additional secondary metabolites, including free amino acids and phytosterols, contribute to the overall dietary value of white maize. The regulation of these pathways occurs primarily at the transcriptional level of biosynthetic enzyme genes and is influenced by both genetic background and environmental cues (Kgoale et al., 2026).

To date, research on yellow maize biofortification and white maize nutritional quality has primarily focused on metabolic pathways and accumulation patterns (Diepenbrock et al., 2021; Luo et al., 2023), genetic regulation (Babu et al., 2013), developmental and environmental factors (Gowda et al., 2015), and the biochemistry and dietary evaluation of nutritional compounds (Pérez-Massot et al., 2013). However, the role of endophytic microorganisms and their metabolites in shaping these distinct nutritional traits remains largely unexplored.

The endophytic microbial community, often regarded as the plant's “second genome,” plays a pivotal role in host growth, development, and defense (Turner et al., 2013). The dynamic interaction between plants and their associated microbial communities involves intricate metabolic exchanges that lead to the selective recruitment and enrichment of endophytes (Bulgarelli et al., 2013). These microorganisms can further modulate the production of plant secondary metabolites (Pang et al., 2021). The plant metabolome, encompassing both primary and secondary metabolites, represents the functional output of genetic and environmental influences (Fiehn, 2002). Integrating microbiome and untargeted metabolomic data offers a powerful approach to unravel how endophytes contribute to plant phenotypic traits, including grain nutrition (Trivedi et al., 2020).

The present work had simultaneously quantified kernel carotenoids, resolved kernel endophytic bacterial and fungal communities by 16S rRNA and ITS amplicon sequencing, and profiled the kernel metabolome in the same yellow and white maize cultivars. Based on these considerations, we hypothesized that yellow and white maize harbor distinct endophytic microbiomes and metabolomes that are associated with their contrasting nutritional phenotypes. This study therefore aimed to compare kernel endophytic communities and metabolic profiles between the two morphotypes in an explicitly observational, association-based framework, and thereby to identify candidate microbes and metabolites as testable hypotheses for future causality studies.

## 2 Materials and methods

### 2.1 Experimental sites and materials

The experimental site was situated in Natao Township, Bama Yao Autonomous County, Hechi City, Guangxi Zhuang Autonomous Region (24.014789° N, 107.334380° E). The plant materials included two yellow maize (H) cultivars: ‘Bama Pearl Yellow (A)’ and ‘C8380 (B)’, as well as two white maize (B) cultivars: ‘Longlin White (C)’ and ‘Guibai waxy (D)’ (See Fig. 1). Four plants were cultivated for each cultivar. All cultivars were planted on July 10, 2023, and managed under identical conventional cultivation practices. During the growth period the site experienced a mean daily air temperature of approximately 26 °C, a mean relative humidity of approximately 76%, a mean daily photosynthetically active radiation of approximately 1250 μmol m^−2^ s^−1^, and a natural day length of approximately 12.5 h; the soil was a sandy loam (pH approximately 6.4) and the field was flood-irrigated twice during the season. No chemical defoliant was applied. Mature ears were hand-harvested in mid-October 2023, approximately 105 days after sowing, at which stage the mean plant height was approximately 2.1 m. Intact kernels were separated from the cobs, surface-cleaned, and air-dried to constant weight in a ventilated drying oven at 26 °C.

**Fig. 1:**
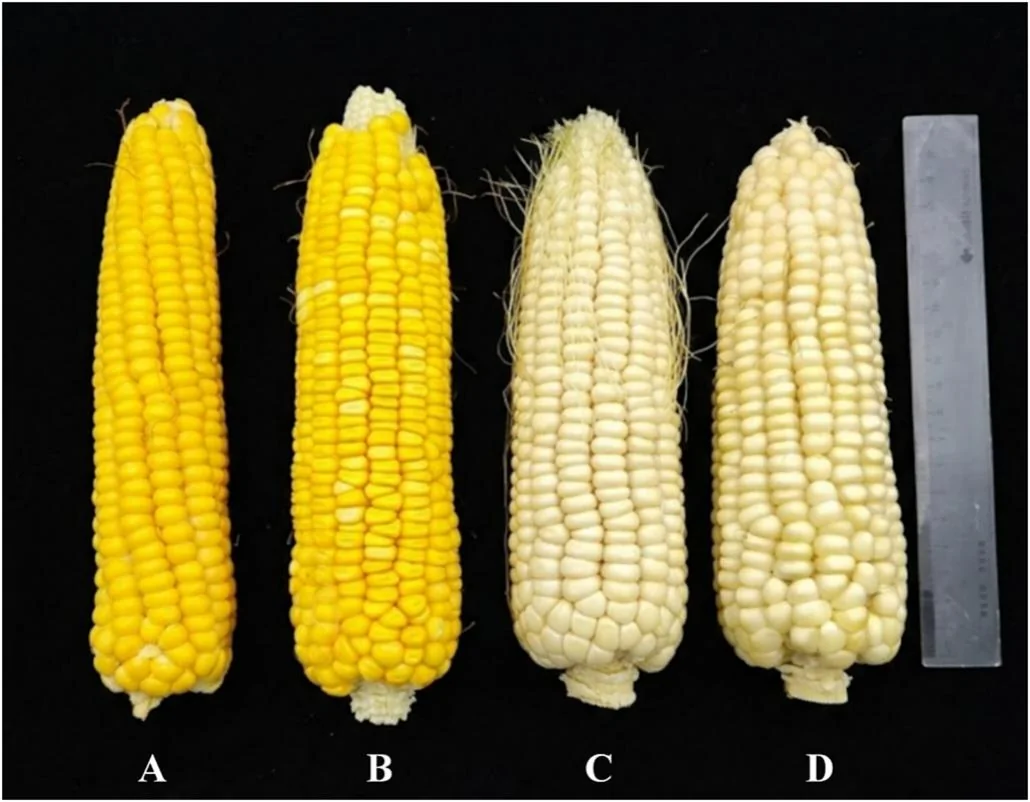
The yellow maize cultivars ‘Bama Pearl Yellow (A)’ and ‘C8380 (B)’, and the white maize cultivars ‘Longlin White (C)’ and ‘Guibai waxy (D)’. (For interpretation of the references to colour in this figure legend, the reader is referred to the web version of this article.)

### 2.2 Chemicals and reagents

Absolute ethanol (analytical reagent, ≥99.7%), potassium hydroxide (≥95.0%), and 2,6-di-tert-butyl-p-cresol (BHT, ≥99.0%) were obtained from Sinopharm Chemical Reagent Co., Ltd. (Shanghai, China). Cyclohexane (≥99.5%), diethyl ether (≥99.5%), n-hexane (HPLC grade, ≥97.0%), anhydrous sodium sulfate (≥99.0%), and *N*,*N*-dimethylformamide (≥99.5%) were purchased from Aladdin Industrial Corporation (Shanghai, China). Methanol, acetonitrile, and methyl tert-butyl ether (all HPLC grade, ≥99.9%) were supplied by Merck KGaA (Darmstadt, Germany). Lutein (≥95.0%, purity determined by HPLC) and zeaxanthin (≥95.0%, purity determined by HPLC) reference standards were purchased from Sigma-Aldrich (St. Louis, MO, USA). Ultrapure water (18.2 MΩ·cm) was produced in-house with a Milli-Q water purification system (Merck Millipore, Billerica, MA, USA). All other chemicals used for endophytic microbiome analysis, including the FastDNA® Spin Kit for Soil (MP Biomedicals, Solon, OH, USA) and the AxyPrep DNA Gel Extraction Kit (Axygen Biosciences, Union City, CA, USA).

Agarose, agar, tryptone, yeast extract, and sodium chloride for lysogeny broth (LB) medium were obtained from Oxoid Ltd. (Basingstoke, UK). Potato dextrose agar (PDA) was obtained from Beijing Aoboxing Biotechnology Co., Ltd. (Beijing, China). Sodium hypochlorite solution (available chlorine ≥5.2%, *w*/*v*) was purchased from Sinopharm Chemical Reagent Co., Ltd. (Shanghai, China) and freshly diluted with sterile distilled water to the working concentration before use.

### 2.3 Surface sterilization and verification of kernel endophytic origin

Prior to microbiome and metabolomic analyses, intact maize kernels were surface-sterilized to remove epiphytic microorganisms and to ensure that the recovered communities represented true endophytes rather than surface contaminants (Hallmann et al., 1997; Schulz & Boyle, 2005). Briefly, twenty kernels per cultivar were randomly selected, rinsed three times with sterile distilled water, immersed in 70% (*v*/v) ethanol for 1 min, transferred to 2.5% (*w*/*v*) sodium hypochlorite for 5 min, rinsed again in 70% (*v*/v) ethanol for 30 s, and finally washed five times with sterile distilled water under aseptic conditions. The 100 μL of the final rinse water was spread onto LB agar and PDA agar plates and incubated at 28 °C for 72 h; no colony growth was observed on either medium, confirming the effective removal of surface-associated microbes. Sterilized maize grains were then ground in liquid nitrogen, and the resulting powders were subsampled in parallel for carotenoid determination, untargeted metabolomic profiling, and endophytic microbiome analysis.

### 2.4 Determination of zeaxanthin and lutein contents

#### 2.4.1 Sample extraction and saponification

Approximately 1.00 g of homogenized maize sample was weighed on an electronic analytical balance (0.1 mg readability; model FA2004, Shanghai Liangping Instruments Co., Ltd., Shanghai, China) and mixed with 6 mL of absolute ethanol containing 1% (*w*/*v*) 2,6-di-tert-butyl-p-cresol (BHT). The mixture was ultrasonicated for 30 min, followed by incubation in a water bath at 70 °C for 30 min. For saponification, 5 mL of 1 mol/L potassium hydroxide (KOH) solution was added. After thorough mixing, the homogenate was saponified in a water bath at 55 °C for 45 min, then immediately cooled in an ice-water bath to terminate the reaction.

#### 2.4.2 Solvent extraction

To extract the carotenoids, 15 mL of an extraction solvent comprising cyclohexane, diethyl ether, and n-hexane (1:2:2, *v*/v/v) containing 0.1% (*w*/*v*) BHT was added to the cooled saponified mixture. The mixture was shaken vigorously for 3 min and allowed to stand until phase separation was complete. The upper organic phase was carefully transferred to a clean centrifuge tube. The remaining aqueous phase was re-extracted twice using the same procedure, and all organic phases were combined. The pooled organic extract was dehydrated with 0.1 g of anhydrous sodium sulfate, filtered, and evaporated to near dryness under a gentle stream of nitrogen. The residue was reconstituted in methanol containing 0.1% (*w*/*v*) BHT to a final volume of 10 mL. The resulting solution was filtered through a 0.22 μm hydrophilic nylon membrane syringe filter (13 mm diameter, 0.22 μm pore size; Jinteng Experimental Equipment Co., Ltd., Tianjin, China) prior to analysis.

#### 2.4.3 High-performance liquid chromatography (HPLC) analysis

Chromatographic analysis was performed on an Agilent 1100 HPLC system (Agilent Technologies Inc., Santa Clara, CA, USA) equipped with a UV–Vis detector set at 445 nm. Separation was achieved on a Compass C30 reversed-phase column (250 mm × 4.6 mm, 5 μm particle size; Bonna-Agela Technologies Inc., Tianjin, China) at 30 °C. The mobile phase consisted of acetonitrile (A) and methyl tert-butyl ether (B), delivered at a flow rate of 1.0 mL/min. The injection volume was 10 μL. Gradient elution was performed as follows: 0 min, 100% A; 15 min, 55% A and 45% B.

#### 2.4.4 Standard curves and quantification

Reference standards of lutein and zeaxanthin were weighed, dissolved in *N*,*N*-dimethylformamide (DMF), and serially diluted with methanol containing 0.1% (*w*/*v*) BHT to obtain six calibration levels of 0.5, 1.0, 5.0, 10.0, 50.0, and 100.0 μg/mL for each standard. These solutions were analyzed under the same chromatographic conditions. Calibration curves were constructed by plotting peak areas (y-axis) against concentrations (x-axis). The contents of lutein and zeaxanthin in the maize cultivars were quantified based on the corresponding calibration curves

### 2.5 Untargeted metabolomic profiling of maize kernels

Approximately 50 mg of each kernel sample was weighed into a 2 mL centrifuge tube. Tissues were homogenized using a cryogenic tissue grinder (MM 400 mixer mill; Retsch GmbH, Haan, Germany) at −10 °C and a frequency of 50 Hz for 6 min, followed by ultrasonic extraction in a temperature-controlled ultrasonic cleaner (KQ-500DE numerical-control ultrasonic cleaner; Kunshan Ultrasonic Instruments Co., Ltd., Jiangsu, China) at −5 °C and 40 kHz for 30 min. The homogenates were then incubated at −20 °C for 30 min to promote protein precipitation, and subsequently centrifuged at 13,000 ×*g* for 15 min at 4 °C in a refrigerated centrifuge (model 5424R; Eppendorf AG, Hamburg, Germany). The clear supernatant was transferred to an LC-MS injection vial. To monitor system stability and ensure data reproducibility, aliquots (20 μL each) from all sample supernatants were pooled to prepare a quality control (QC) sample.

LC-MS analysis. Untargeted metabolomic profiling was performed using a Thermo Scientific ultra-high-performance liquid chromatography (UHPLC) system coupled to a Q Exactive™ Hybrid Quadrupole-Orbitrap™ mass spectrometer (Thermo Fisher Scientific, Waltham, MA, USA). Chromatographic separation was achieved on a Waters ACQUITY UPLC HSS T3 column (100 mm × 2.1 mm, 1.8 μm particle size) maintained at 40 °C. The mobile phase consisted of solvent A (95% water, 5% acetonitrile, and 0.1% formic acid, *v*/v/v) and solvent B (47.5% acetonitrile, 47.5% isopropanol, 5% water, and 0.1% formic acid, *v*/v/*v*/v). The flow rate was set to 0.40 mL/min, and the injection volume was 2 μL. Initial multivariate data analysis was performed using the Majorbio Cloud Platform (https://cloud.majorbio.com).

### 2.6 Endophytic microbiome profiling of maize kernels

Total genomic DNA was extracted from the samples using the FastDNA® Spin Kit (MP Biomedicals, USA) according to the manufacturer's protocol. The concentration and purity of the extracted DNA were assessed using a NanoDrop 2000 spectrophotometer (Thermo Fisher Scientific, USA). PCR amplification was subsequently conducted on an ABI GeneAmp® 9700 thermal cycler (Applied Biosystems, USA). The specific primers and target regions used for amplifying the endophytic bacterial and fungal communities are detailed in Table 1 (Gardes & Bruns, 1993; Klindworth et al., 2013). The resulting PCR amplicons were separated by 2% agarose gel electrophoresis, excised, and purified using the AxyPrep DNA Gel Extraction Kit (Axygen Biosciences, USA).

**Table 1:** Target regions and primer sequences for high-throughput sequencing.

Sequencing Type	Primer Name	Primer Sequence	Sequencing Length	Sequencing platform
Endophytic bacteria	799F	5′-AACMGGATTAGATACCCKG-3′	394 bp	Miseq PE300
	1193R	5′-ACGTCATCCCCACCTTCC-3′		

Endophytic fungi	ITS1F	5′-CTTGGTCATTTAGAGGAAGTAA-3′	350 bp	MiSeq PE300
	ITS2R	5′-GCTGCGTTCTTCATCGATGC-3′		

Purified amplicons from the same sample were pooled in equimolar amounts and quantified using a Quantus™ Fluorometer (Promega, Madison, WI, USA). Amplicon libraries were then constructed using the NEXTFLEX® Rapid DNA-Seq Kit. High-throughput paired-end sequencing was performed on an Illumina MiSeq platform by Shanghai Majorbio Bio-Pharm Technology Co., Ltd. (Shanghai, China).

### 2.7 Weighted gene co-expression network analysis (WGCNA) of metabolites

WGCNA was performed on the metabolite abundance matrix containing differential metabolites from four cultivars (798 metabolites in total) using the Majorbio Cloud platform ([https://analysis.majorbio.com/](https://analysis.majorbio.com/)). A signed network was constructed with a soft-power *β* of 10. Pearson correlation was adopted to calculate pairwise metabolite correlations. The module-merging threshold was set to 9, merge cut height to 0.25, and the kME cutoff to 0.7. For network visualization, the top 30 nodes with the highest intra-module connectivity were retained, and only edges with weight values greater than 0.02 were preserved. Briefly, the correlation matrix was weighted to generate an adjacency matrix, which was further converted into a topological overlap matrix (TOM) for hierarchical clustering, module identification and merging.

### 2.8 Statistical analyses

All experimental data were analyzed using Excel 2021 and SPSS 26.0 and are expressed as the mean ± standard deviation (SD). The scope of application of each statistical procedure was as follows. Lutein and zeaxanthin contents among the four cultivars (Table 2) and the alpha-diversity indices of kernel-endophytic bacteria and fungi between group H and group B (Table 3) were compared by one-way analysis of variance followed by Duncan's multiple range test; differences were considered significant at *P* < 0.05 (denoted by lowercase letters) and highly significant at *P* < 0.01 (denoted by capital letters). Wilcoxon rank-sum tests were applied to the non-normally distributed relative-abundance profiles of individual microbial taxa when comparing group H and group B, and to the genus-level abundance vectors used in the microbial–metabolite correlation analysis. Differential metabolite screening was based on the OPLS-DA model with the thresholds variable importance in the projection (VIP) > 1 and *P* < 0.05. To control the false discovery rate across the large number of simultaneous metabolite-level and taxon-level tests, Benjamini–Hochberg FDR correction was applied to (i) the metabolite-level *P* values from the differential accumulation analysis, (ii) the *P* values supporting the LEfSe biomarker assignments, and (iii) the *P* values of the microbial–metabolite correlation matrices. Bacterial and fungal community composition and functional predictions were performed using the I-sanger Cloud Platform (https://www.majorbio.com/web/www/index). Comprehensive bioinformatics and multi-omics data analyses were performed using the Majorbio Cloud Platform (http://www.majorbio.com). Metabolic pathway annotation and topological analyses were conducted utilizing the Kyoto Encyclopedia of Genes and Genomes (KEGG) database (https://www.kegg.jp).

**Table 2:** Contents of lutein and zeaxanthin in yellow and white maize cultivars (mean ± SD) (μg/g).

Cultivars	Lutein	Zeaxanthin
A	5.450 ± 0.380 ^A^	8.014 ± 0.645 ^B^
B	4.737 ± 0.052 ^B^	15.302 ± 0.551 ^A^
C	0.029 ± 0.002^C^	0.109 ± 0.008^C^
D	0.033 ± 0.003^C^	0.059 ± 0.004^C^

Note: Different capital letters within the same column indicate extremely significant differences at *P* < 0.01.

**Table 3:** Alpha diversity analysis of kernel-endophytic microorganisms in maize (mean ± SD).

Classifications	Cultivars	Shannon	Simpson	Ace	Chao **1**	Coverage
kernel-endophytic Bacteria	H	4.179 ± 0.351a	0.041 ± 0.020a	456.663 ± 93.551a	451.641 ± 96.671a	0.999
	B	4.258 ± 0.380a	0.040 ± 0.021a	512.128 ± 159.380a	497.164 ± 152.274a	0.999
						
kernel-endophytic Fungi	H	1.116 ± 0.456a	0.440 ± 0.174b	34.454 ± 11.144a	34.375 ± 10.949a	1.000
	B	0.880 ± 0.531b	0.609 ± 0.233a	47.344 ± 17.108a	46.875 ± 16.891a	1.000

Notes: Data are presented as mean ± SDs. Different lowercase letters in the same column indicate significant differences based on Duncan's multiple range test at *p* < 0.05.

## 3 Results

### 3.1 Lutein and zeaxanthin contents in yellow and white maize cultivars

As shown in Table 2, the contents of lutein and zeaxanthin varied significantly among the four maize cultivars. The yellow maize cultivars, ‘Bama Pearl Yellow’ (A) and ‘C8380’ (B), exhibited significantly higher levels of both carotenoids compared to the white maize cultivars, ‘Longlin White’ (C) and ‘Guibai waxy’ (D). Among the yellow maize cultivars, cultivar A recorded the highest lutein content (5.450 ± 0.380 μg/g), which was significantly greater than that of cultivar B. Conversely, cultivar B possessed a significantly higher zeaxanthin content (15.302 ± 0.551 μg/g) than cultivar A. In contrast, both white maize cultivars contained only trace amounts of these pigments, with no significant differences observed between them.

To further investigate whether these significant differences in carotenoid accumulation influence microbial colonization, the two yellow and two white maize cultivars were categorized into the H and B groups, respectively, for subsequent microbiome and untargeted metabolomic analyses. It should be noted that, in the present design, grain colour and cultivar genotype are not independently manipulated, so colour effects and genotype effects are inherently confounded. The two cultivars within each colour group were therefore treated as biological replicates of a colour-defined group, and all group-level inferences (H vs. B) should be interpreted as differences between grain-colour morphotype groups rather than as effects of kernel colour per se.

### 3.2 Analysis of endophytic microbes in yellow and white maize cultivars

#### 3.2.1 Alpha diversity analysis of kernel-endophytic microorganisms

As presented in Table 3, the coverage rate of all samples was higher than 99.9%, confirming adequate sequencing depth and the high reliability of the acquired microbial community data. For kernel-endophytic bacteria, no significant differences were observed in alpha diversity (Shannon and Simpson indices) and community richness (Ace and Chao1 indices) between group H and group B. However, distinct differences in fungal alpha diversity were detected between the two groups, while no significant variations were found in the community richness of kernel-endophytic fungi. Specifically, group H exhibited a significantly higher Shannon index and a markedly lower Simpson index compared with group B, reflecting a more diverse endophytic fungal community in yellow maize kernels.

#### 3.2.2 Beta diversity analysis of kernel-endophytic bacterial and fungal communities in yellow and white maize cultivars

At the operational taxonomic unit (OTU) level, Partial Least Squares Discriminant Analysis (PLS-DA) revealed clear separations of kernel endophytic bacterial (Fig. 2a) and fungal (Fig. 2b) communities between yellow (H) and white (B) maize. These results indicate that the community compositions of endophytic bacteria and fungi differ significantly between the two cultivars.

**Fig. 2:**
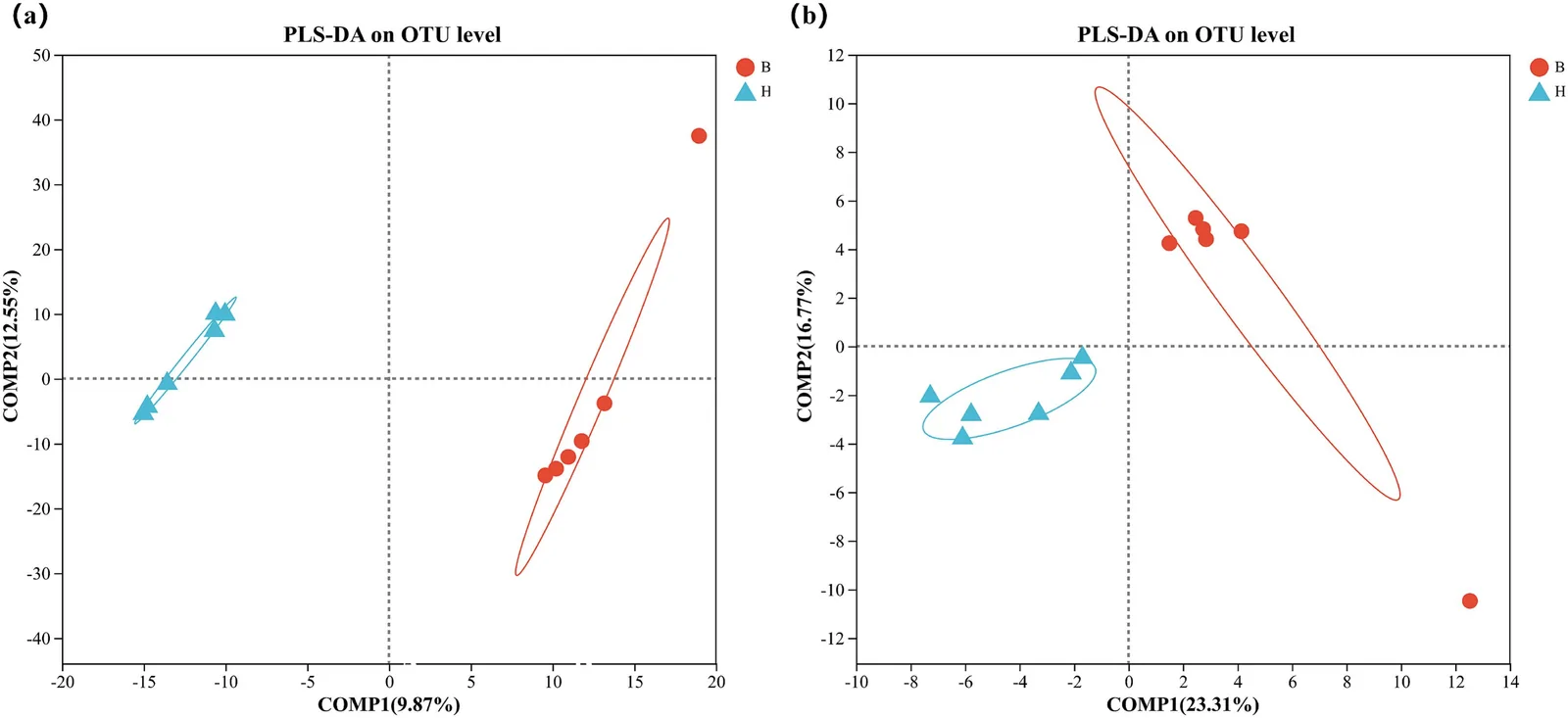
PLS-DA score plots comparing kernel bacterial (a) and fungal (b) communities between yellow (H) and white (B) maize. (For interpretation of the references to colour in this figure legend, the reader is referred to the web version of this article.)

#### 3.2.3 Kernel-endophytic microbial community compositions in yellow and white maize cultivars

At the phylum level, four and six dominant endophytic bacterial phyla (relative abundance >1%) were identified in H and B kernels, respectively. Proteobacteria, Bacteroidota, Actinobacteriota, and Firmicutes were the dominant phyla shared by both H and B. In contrast, Chloroflexi and Calditrichota were unique dominant phyla exclusive to B. Notably, Proteobacteria exhibited the highest relative abundance in both groups, representing the largest proportion of the bacterial communities (Fig. 3a). At the genus level, several dominant endophytic bacterial genera were detected in both H and B kernels. In H, *Tepidiamorphus* and *unclassified_o__Rhizobiales* were among the most abundant genera, whereas *Pseudomonas* was the most abundant genus in B. In comparison with B, unc*lassified_o__Rhizobiale*s, and *Wenzhouxiangella* were uniquely dominant in H kernels. Conversely, *norank_f__Rikenellaceae*, *unclassified_f__Oxalobacteraceae*, *norank_f__norank_o__Microtrichales*, and *unclassified_p__Chloroflexi* were uniquely dominant in B kernels (Fig. 3b).

**Fig. 3:**
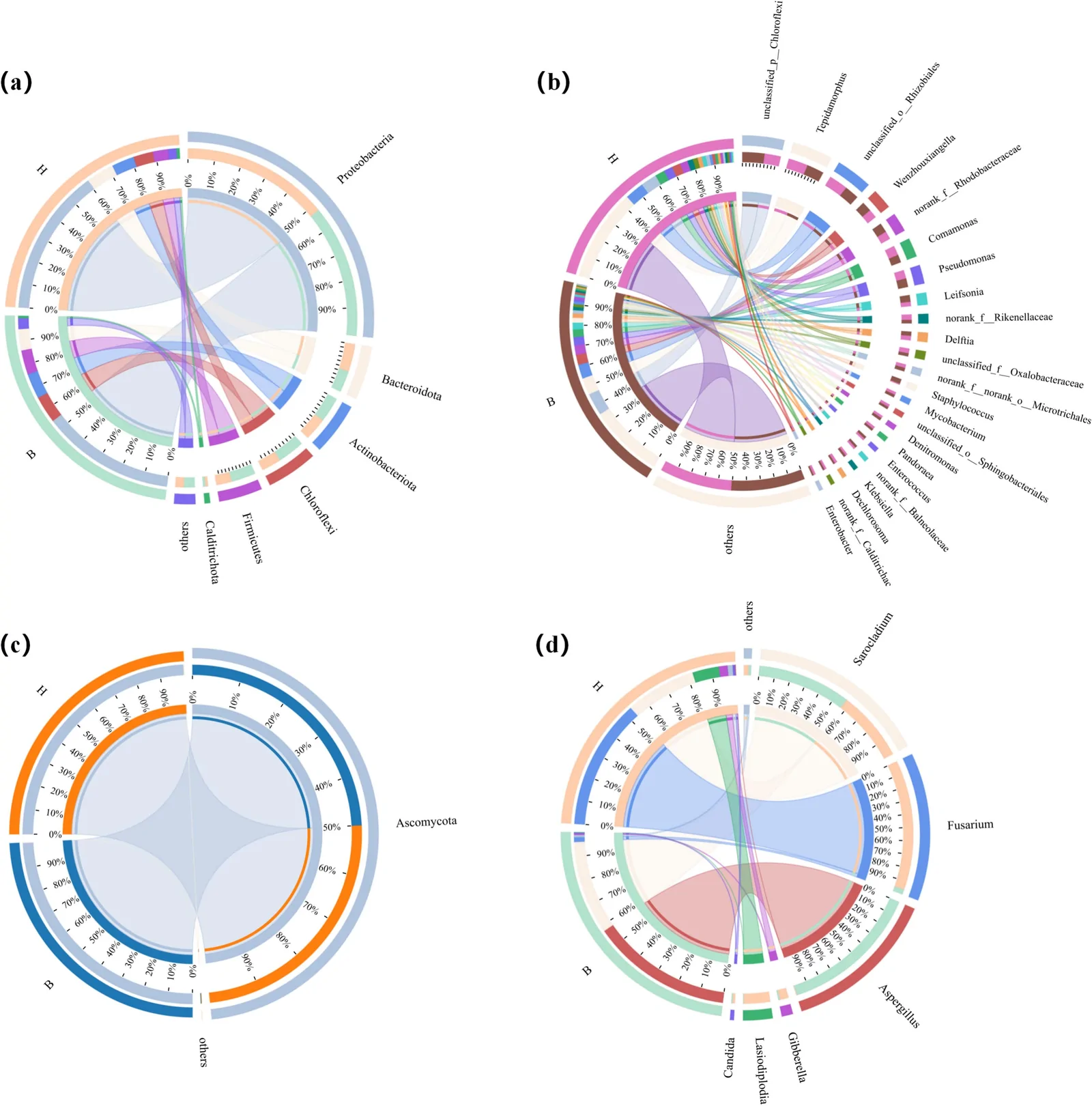
Composition of kernel-endophytic microbial communities in yellow (H) and white (B) maize. Bacterial communities at the phylum (a) and genus levels (b); fungal communities at the phylum (c) and genus levels (d). (For interpretation of the references to colour in this figure legend, the reader is referred to the web version of this article.)

Regarding fungi, Ascomycota was the overwhelmingly dominant phylum in both H and B, accounting for nearly all identified fungal sequences at the phylum level (Fig. 3c). At the genus level, four and six dominant endophytic fungal genera were detected in H and B kernels, respectively. Common dominant genera included *Sarocladium*, *Fusarium*, *Gibberella*, and *Lasiodiplodia*. The mean relative abundance of *Fusarium* and *Lasiodiplodia* appeared higher in *H* than in *B,* whereas *Aspergillus* showed higher abundance in B (Fig. 3d).

Venn diagram analysis further revealed that B and H harbored 40 and 41 kernel-endophytic bacterial phyla, respectively, with 4 and 5 unique phyla in each group (Fig. 4a). At the genus level, 629 and 600 bacterial genera were identified in B and H harbored, respectively, including 171 and 142 unique genera (Fig. 4b).

**Fig. 4:**
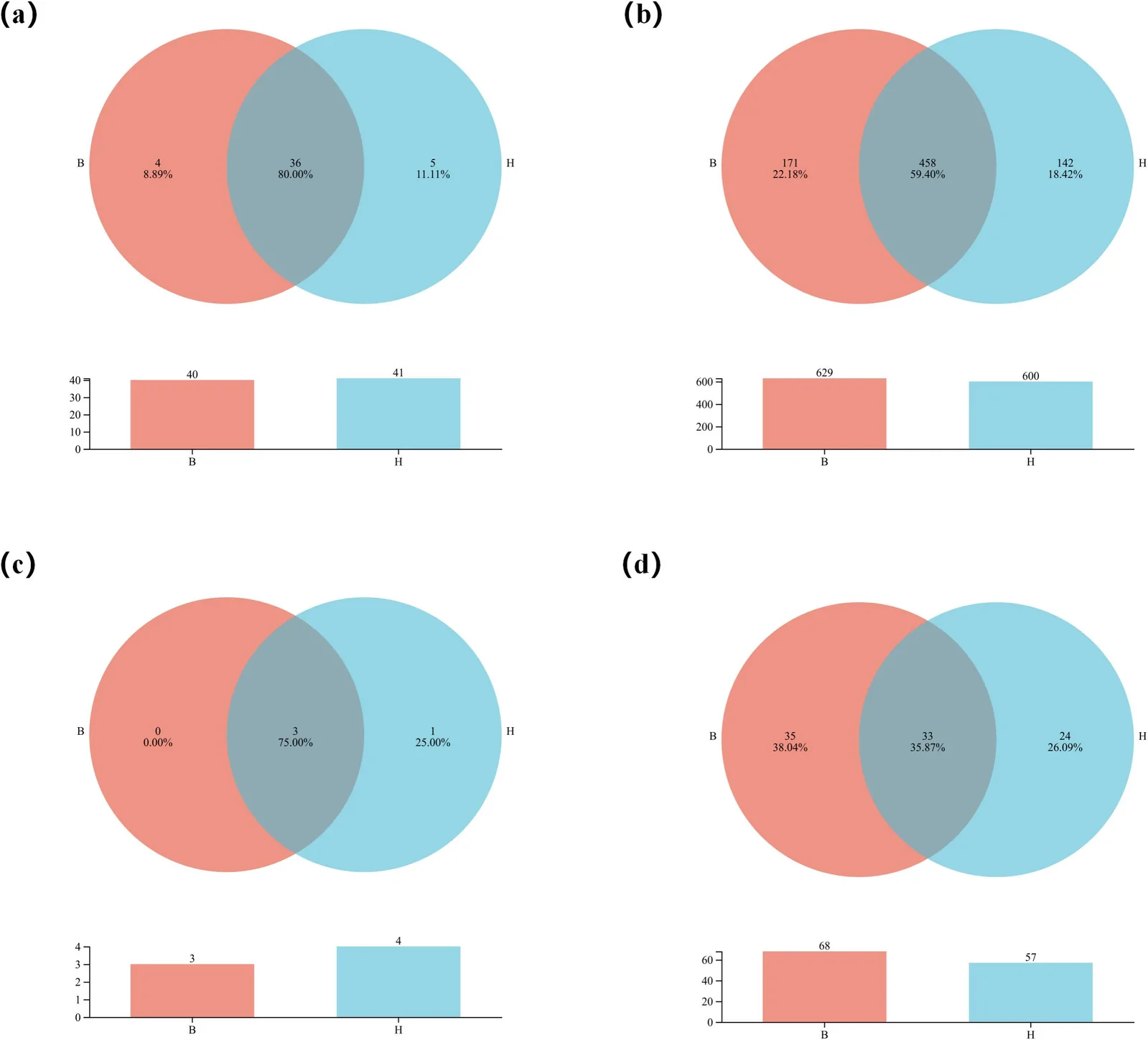
Venn diagram analysis of kernel-endophytic bacteria at the phylum (a) and genus (b) levels, and fungi at the phylum (c) and genus (d) levels.

For fungi, three and four phyla were identified in B and H kernels, respectively, with no unique phylum in B and one unique phylum in H (Fig. 4c). At the genus level, B and H contained 68 and 57 fungal genera, respectively, including 35 and 24 unique genera (Fig. 4d).

#### 3.2.4 LEfSe analysis of endophytic bacterial and fungal communities in yellow and white maize cultivars

Linear Discriminant Analysis Effect Size (LEfSe, LDA score > 1.0) analysis was performed to identify significantly differential biomarkers in kernel-endophytic communities between yellow (H) and white (B) maize cultivars.

As shown in Fig. 5a, Proteobacteria was significantly enriched in H kernels at the phylum level. At the genus level, *Bdellovibrio*, *Neisseria*, *Acidaminobacter*, and *Syntrophobacter* were significantly enriched in H. In contrast, *Aerococcus*, *Moraxella*, *Corynebacterium*, and *Peredibacter* were significantly aggregated in B kernels. It should be noted that *Tepidiamorphus*, although among the most abundant genera in H kernels (Fig. 3b), was not retained as a significant bacterial biomarker by the LEfSe analysis and is therefore treated throughout this study as a dominant genus identified by community-composition profiling rather than as a statistically supported differential biomarker.

**Fig. 5:**
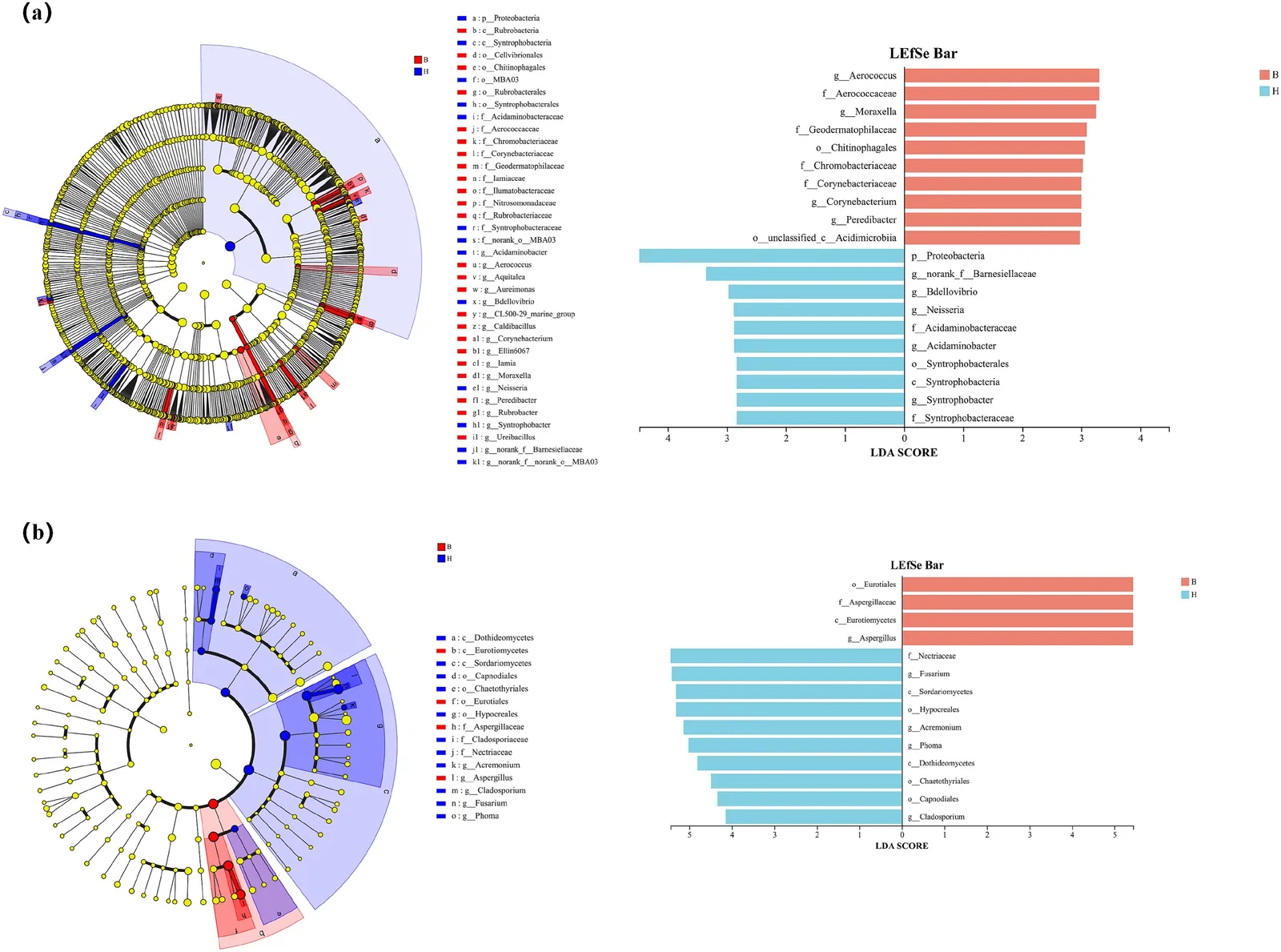
LEfSe analysis of kernel-endophytic bacteria (a) and fungi (b) in yellow (H) and white (B) maize cultivars. The cladograms (left) display the phylogenetic distribution of differentially abundant taxa, and the LDA score bar plots (right) show the biomarkers with LDA scores >1.0. Red and blue indicate enrichment in H and B, respectively. (For interpretation of the references to colour in this figure legend, the reader is referred to the web version of this article.)

For endophytic fungi (Fig. 5b), 10 and 4 clades were identified as significant biomarkers in H and B kernels, respectively. At the class level, Dothideomycetes and Sordariomycetes were significantly enriched in H kernels, whereas Eurotiomycetes was significantly enriched in B kernels. At the genus level, *Fusarium*, *Acremonium*, *Phoma*, and *Cladosporium* were significantly enriched in H kernels. In contrast, *Aspergillus* was significantly aggregated in B kernels.

#### 3.2.5 Functional predictive analysis of endophytic bacterial and fungal communities in yellow and white maize cultivars

The functional traits of kernel-endophytic bacteria in H and B maize cultivars were predicted using BugBase. Both groups exhibited similar bacterial phenotypic categories, namely Gram_Negative, Aerobic, Forms_Biofilms, Stress_Tolerant, Potentially_Pathogenic, Facultatively_Anaerobic, Gram_Positive, Contains_Mobile_Elements, and Anaerobic. Overall, the relative abundances of these phenotypic categories were comparable between the two groups, indicating a stable functional structure in the kernel bacterial communities (Fig. 6a).

**Fig. 6:**
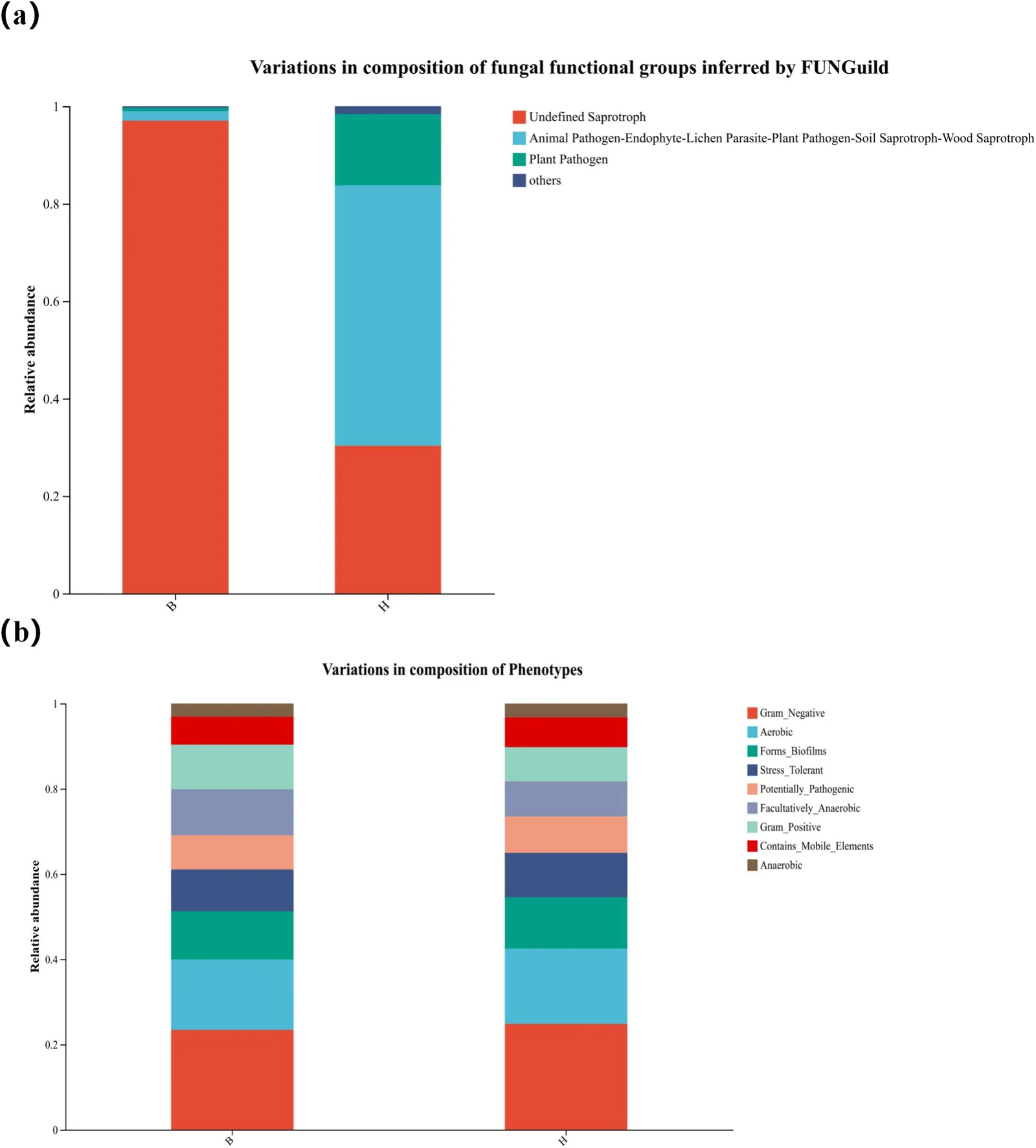
Functional predictions of endophytic bacterial (a) and fungal (b) communities in yellow and white maize cultivars. (For interpretation of the references to colour in this figure legend, the reader is referred to the web version of this article.)

The ecological guilds of kernel-endophytic fungi were further predicted using FUNGuild. The primary functional guilds identified included Undefined Saprotroph, Animal Pathogen-Endophyte-Lichen Parasite-Plant Pathogen-Soil Saprotroph-Wood Saprotroph, and Plant Pathogen. Notably, the composition of these fungal guilds differed markedly between the two cultivars. In B kernels, Undefined Saprotroph was the overwhelmingly dominant guild, accounting for the vast majority of the community. In contrast, H kernels showed a substantially reduced relative abundance of Undefined Saprotroph, coupled with significant enrichment of the multi-trophic guild and Plant Pathogen (Fig. 6b).

### 3.3 Analysis of metabolites in yellow and white maize cultivars

Metabolites profiles of maize kernels from yellow and white cultivars were systematically analyzed via principal component analysis (PCA) and orthogonal partial least squares discriminant analysis (OPLS-DA). The PCA results showed that the first two principal components (PC1 and PC2) explained 26.20% and 14.10% of the total phenotypic variance, respectively (Fig. 7a). For the OPLS-DA model, the first component and the first orthogonal component accounted for 13.50% and 24.70% of the total variance separately (Fig. 7b). Consistent clustering patterns were observed in both models, where kernel metabolites of yellow (H) and white (B) maize formed completely independent clusters with clear intergroup separation. These results demonstrated significant differences in metabolic profiles between the two maize cultivars.

**Fig. 7:**
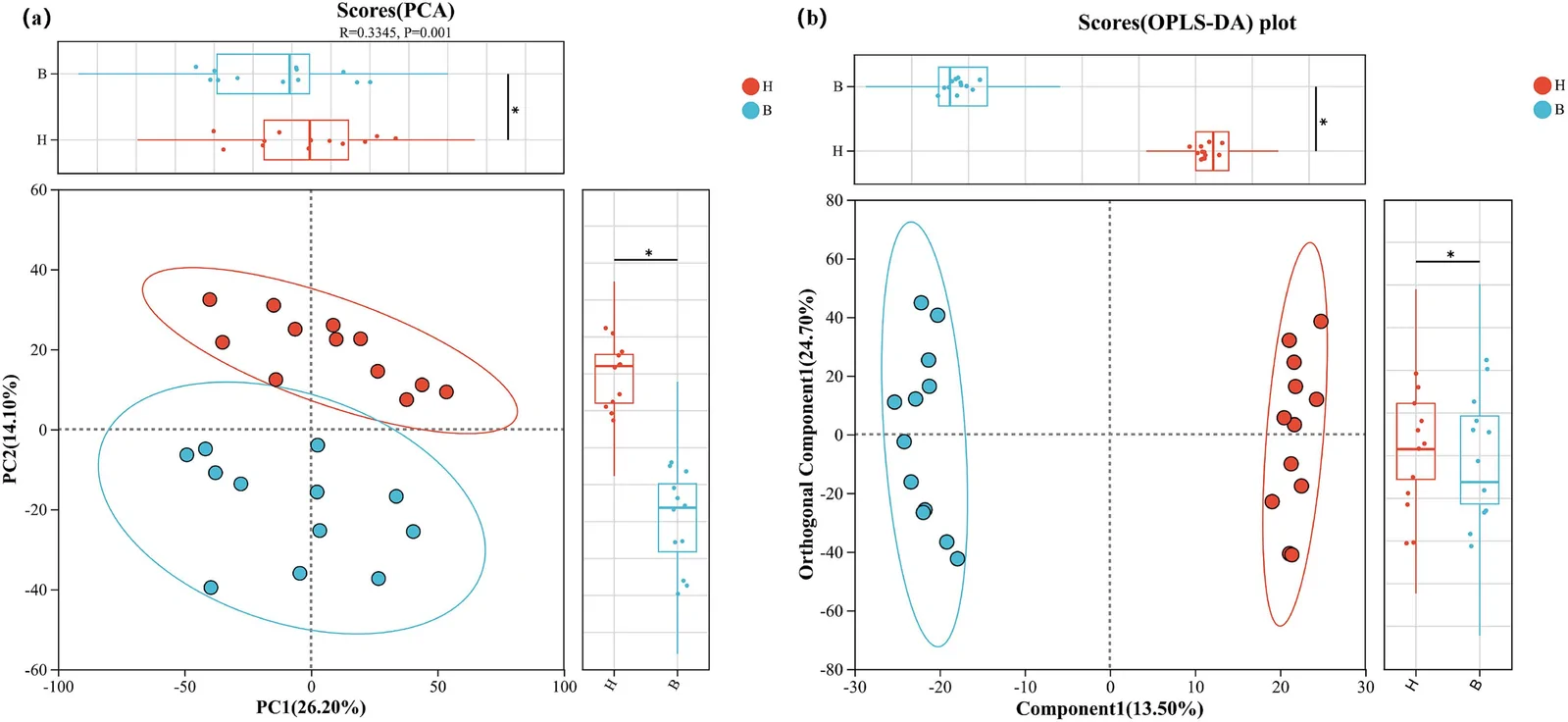
PCA (a) and OPLS-DA (b) score plots of kernel metabolites from yellow and white maize cultivars. H: yellow maize cultivar; B: white maize cultivar. (For interpretation of the references to colour in this figure legend, the reader is referred to the web version of this article.)

Significant differential metabolites were screened based on the combined thresholds of *p* < 0.05 and variable importance in the projection (VIP) > 1 derived from the OPLS-DA model. A total of 313 significantly differentially metabolites were identified between the two cultivars. Specifically, compared with white maize (B), 79 metabolites were significantly upregulated, while 236 metabolites were significantly downregulated in the kernels of yellow maize (H) (Fig. 8a). All identified differentially accumulated metabolites (DMs) were further annotated and classified according to the Human Metabolome Database (HMDB), and were grouped into several major superclasses (Fig. 8b). The dominant metabolite categories shared by both cultivars included lipids and lipid-like molecules, organic acids and derivatives, organic oxygen compounds, organoheterocyclic compounds, as well as phenylpropanoids and polyketides. Further refined classification of specific compounds revealed extensive diversity in secondary metabolites, with prominent enrichment of monoterpenoids, monolignols, flavonoids, and multiple classes of alkaloids (Fig. 8c).

**Fig. 8:**
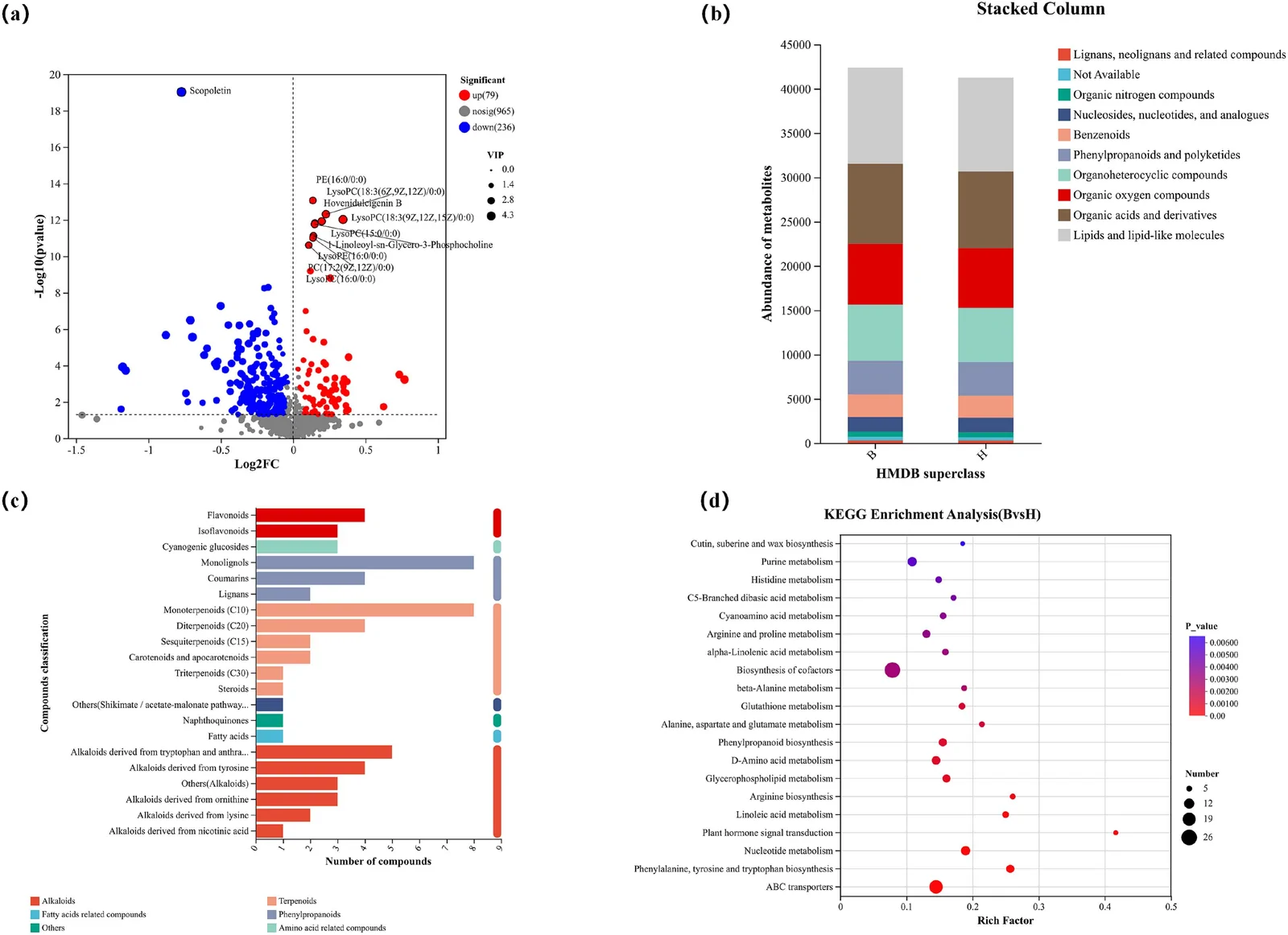
(a) volcano plots of differential metabolites in the H vs. B comparison group. (b) HMDB-based superclass classification of differential metabolites. (c) Detailed compound classification of differential metabolites. (d) KEGG pathway enrichment analysis of identified differential metabolites.

Compound annotation confidence was reported following the Metabolomics Standards Initiative (MSI) guidelines (Blaženović et al., 2018; Sumner et al., 2007). Because our annotation workflow relied on accurate-mass matching and MS/MS spectral similarity against public databases, all DMs were assigned at Level 2 (putatively annotated compounds); none of them was confirmed against an authenticated reference standard analyzed in-house and therefore none reached Level 1 (confirmed structure). A small number of annotations corresponded to pharmaceutical or otherwise biologically implausible compounds in maize kernels, such as sildenafil, penciclovir, and retaspimycin (Table S1). These features were retained in the annotation table for transparency but were re-classified as Level 3 (putatively characterized compound classes), interpreted as probable exogenous contamination or MS/MS library misannotation rather than as endogenous maize metabolites (Blaženović et al., 2018), and excluded from all pathway-enrichment, co-expression-network, and microbial–metabolite correlation analyses. No biological conclusion in this study relies on any of these compounds.

Differential metabolites were additionally filtered by Benjamini–Hochberg false discovery rate (FDR) correction to limit the expected proportion of false positives arising from the large number of simultaneous metabolite-level tests.

KEGG pathway enrichment analysis was subsequently performed to explore the functional metabolic pathways between the kernels of H and B (Fig. 8d). Particularly, several key pathways exhibited significant enrichment, including ABC transporters, phenylalanine, tyrosine, and tryptophan biosynthesis, plant hormone signal transduction, and nucleotide metabolism. These findings indicated that yellow and white maize cultivars possess distinct active metabolic networks and underlying regulatory mechanisms during kernel development.

### 3.4 Analysis of different corn-specific metabolites

whereas the WGCNA input matrix comprised 798 metabolites that passed quality filtering and showed variation across the four individual cultivars (A–D), and the broader 798-metabolite matrix was used because it preserved cultivar-level resolution. The expression profile and VIP analysis (Fig. 9a) revealed cultivar-specific accumulation patterns, and metabolites with VIP > 2 were designated as key biomarkers for each cultivar. Key metabolites for each cultivar are highlighted: A (yellow) enriched in adenylates, B (yellow) in lysophosphatidylcholines, C (white) in terpenoids or alkaloids, D (white) in organic acids and acylamino acids. To dissect the metabolic basis of grain-colour divergence in maize, we performed weighted gene co-expression network analysis (WGCNA) on 798 differentially accumulated metabolites across four cultivars: the yellow-grained Bama Pearl Yellow (A) and C8380 (B), and the white-grained Longlin White (C) and Guibai waxy (D). Four cultivar-specific co-expression modules were identified and mapped to individual cultivars: MElightgreen (A), MEpurple (B), MEmidnightblue (C), and MEgreen (D) (Fig. 9b, c). Yellow and white maize showed a clear bifurcation in module composition: the key modules of yellow cultivars were enriched in phospholipid and nucleotide metabolism, whereas those of white cultivars were dominated by terpenoids, alkaloids, and phenolic acids. Module-trait correlation analysis (Fig. 9c) confirmed that each key module was most positively correlated with its cognate cultivar but negatively correlated with the white group, indicating opposing metabolic reprogramming between the two grain-colour types. We then constructed a co-occurrence network of the four modules (Fig. 9d, Table S2), in which MEgreen, MElightgreen, MEmidnightblue, and MEpurple comprised 20, 9, 20, and 20 nodes, accounting for 28.99%, 13.04%, 28.99%, and 28.99% of the network, respectively. Integrating connectivity data from Table S2 and applying a Degree >2 filter retained 67 highly connected metabolites (two nodes with Degree ≤ 2 excluded): module A (MElightgreen) was represented by adenylates (Adenosine 3′-monophosphate, Adenosine 5’-Monophosphate), module B (MEpurple) by lysophosphatidylcholines (Hovenidulcigenin B, 1-Linoleoyl-sn-Glycero-3-Phosphocholine), module C (MEmidnightblue) by terpenoids and alkaloids ((+/−)-(Z)-2-(5-Tetradecenyl)cyclobutanone, D-(+)-dihydrocarvone), and module D (MEgreen) by organic acids and acylamino acids (3-Hydroxybenzoic Acid, N-Oleoyl Glycine). The VIP > 2 metabolites overlapped substantially with the core nodes of their corresponding modules. For example, Hovenidulcigenin B in cultivar B (connectivity 13.09) and 3-Hydroxybenzoic Acid in cultivar D (13.47) exhibited both high discriminant power and high network centrality, validating the module assignment.

**Fig. 9:**
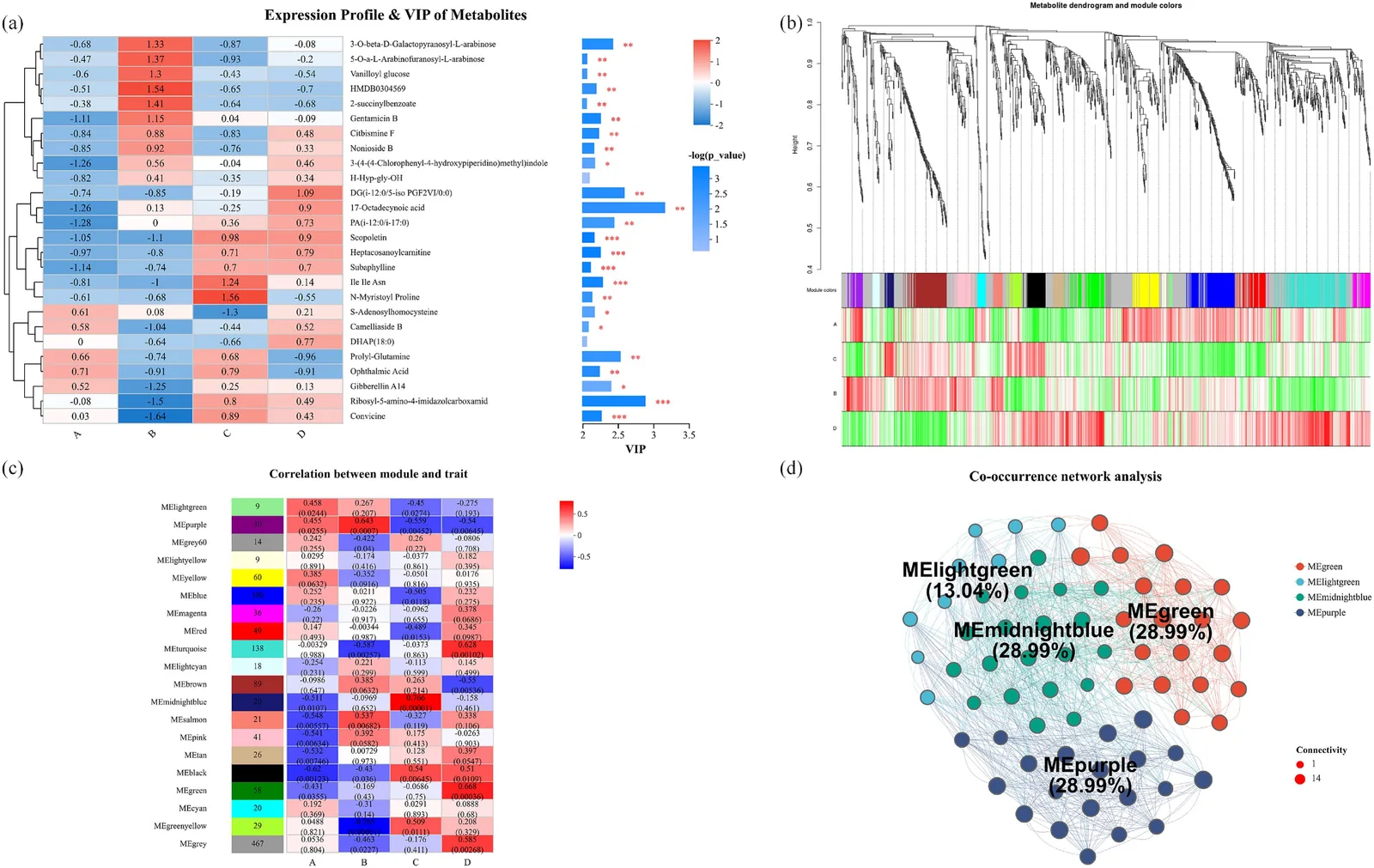
Integrative analysis of metabolomic profiles, WGCNA module identification, and module-trait correlations across four maize cultivars. (a) Expression Profile & VIP of Metabolites. Hierarchical heatmap of standardized relative abundances of key metabolites across cultivars A-D (left); VIP scores and significance are shown as horizontal bars (right; bar colour indicates -log10(P), *P < 0.05, **P < 0.01, ***P < 0.001). (b) Metabolite dendrogram and module colors. Clustering tree of 798 differential metabolites with dynamic branch cutting; MElightgreen, MEpurple, MEmidnightblue, and MEgreen represent the key modules for A, B, C, and D, respectively. (c) Correlation between module and trait. Pearson correlation between module eigengenes and the four cultivars (colour scale −0.5 to 0.5; *P* values in parentheses); each key module shows the strongest positive correlation with its corresponding cultivar. (d) Co-occurrence network analysis. Network constructed from the four cultivar-key modules (MEgreen maps to D, MElightgreen to A, MEmidnightblue to C, MEpurple to B); node colour denotes module or cultivar, node size reflects connectivity, edges denote co-occurrence, and numbers indicate node counts and percentages.

### 3.5 Integrated microbiome–metabolome analysis of yellow and white maize kernels

To dissect the potential links between kernel-associated microorganisms and metabolic phenotypes in yellow and white maize, we performed Pearson correlation analysis between group-mean abundance profiles of bacterial genera (*n* = 24), fungal genera (*n* = 10), and metabolites (*n* = 798) across the four treatment groups (A–D). Hierarchical clustering (complete-linkage, Euclidean distance) was applied to both axes of the correlation matrices to identify coherent association modules.

The bacterium–metabolite correlation matrix (Fig. 10a) revealed two sharply contrasting modules. One module comprised *Staphylococcus*, *Skermania*, *Dietzia*, *Shewanella*, and *Micropruina*, which collectively showed strong positive correlations (*P* < 0.001) with a defined set of metabolites, including goshonoside F1, muscarine, N-(3-hydroxypropyl)valine, episol-tocopherol, hesperetin, malate, and 3-hydroxy-beta-ionone. In direct opposition, a second module consisting of *norank_f_norank_o_nora*, *Taonella*, *Paramaledivibacter*, *norank_f_NS11-12_marine_group*, and *Marinobacter* exhibited equally strong negative correlations (*P* < 0.001) with these same metabolites. A third cluster of taxa—including *Solirubrobacter*, *Novosphingobium*, *Mycobacterium*, *Asticcacaulis*, and *Rhodobacter*—displayed an inverse pattern, correlating positively with an alternative metabolite suite that included neferine, gibberellin A14, asperulosidic acid, vanilloyl glucose, adenosine 5′-monophosphate, and 5-(dimethylamino)liduridine while showing negative associations with the first metabolite group.

**Fig. 10:**
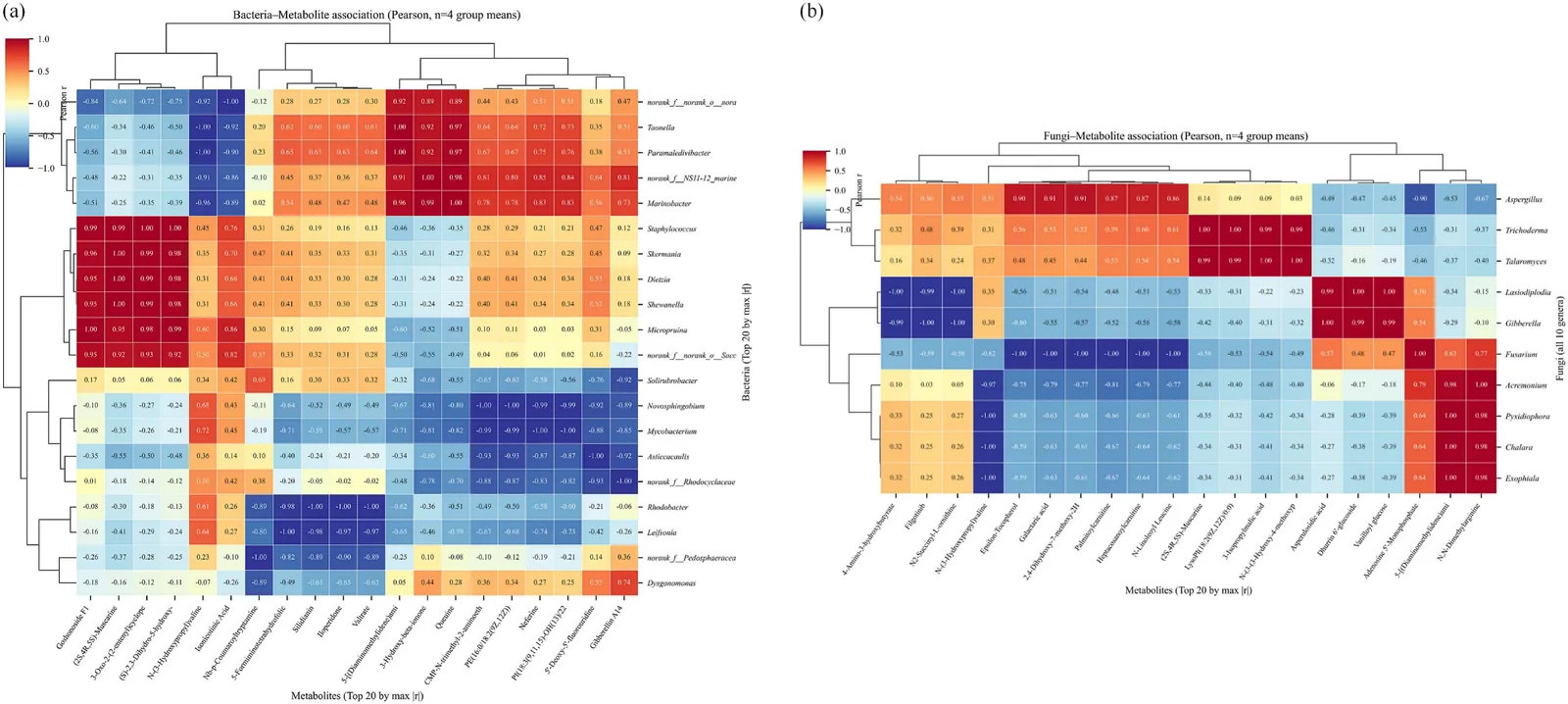
Heatmaps illustrating the correlations between microorganisms and metabolites in yellow corn kernels versus white corn kernels (a) Heatmap showing the correlation between metabolites and bacterial genus-level abundance (b) Heatmap demonstrating the correlation between metabolites and fungal genus-level abundance. (For interpretation of the references to colour in this figure legend, the reader is referred to the web version of this article.)

The fungus–metabolite correlation matrix (Fig. 10b) displayed a similarly modular architecture but with fewer genera. *Aspergillus*, *Trichoderma*, and *Talaromyces* formed a positive-correlation cluster associated primarily with amino-acid-derived and small-molecule metabolites such as 4-amino-3-hydroxybutyrate, filigustinin, N2-succinyl-L-ornithine, palmitoylcarnitine, heptacosanoylcarnitine, galactaric acid, N-linoleoyl leucine, and lysoPI(18:2(9Z,12Z)/0:0). By contrast, *Lasiodiplodia* and *Gibberella* constituted a negative-correlation module against most of these metabolites. Notably, *Fusarium*, *Acremonium*, *Pyxidiophora*, *Chalara*, and *Exophiala* occupied intermediate positions in the dendrogram, each displaying genus-specific correlation patterns that did not align cleanly with either major module. The strongest single association observed was between Trichoderma and (2*S*,4*R*,5*S*)-muscarine (*P* < 0.001).

## 4 Discussion

Maize is one of the most important cereal crops worldwide, accumulating abundant nutrients and bioactive secondary metabolites. It is particularly rich in carotenoids, including zeaxanthin and lutein, which are essential for human eye health. Since humans are incapable of synthesizing these compounds endogenously and must acquire them via dietary intake or supplements, maize serves as a primary natural source of these two functional carotenoids (Eggersdorfer & Wyss, 2018; Krinsky & Johnson, 2005). In yellow maize, kernel coloration and nutritional value are predominantly determined by the accumulation of zeaxanthin and lutein. Meta-analyses have demonstrated substantial variations in total carotenoid content across maize cultivars, with zeaxanthin and lutein levels acting as the primary indicators of nutritional quality (Diepenbrock et al., 2021). Provitamin A and other lipophilic metabolites also contribute greatly to the nutritional biofortification of maize grains (Sun et al., 2022). In contrast, the unique nutritional characteristics of white maize are governed by a distinct set of secondary metabolites. Recent metabolomic studies have confirmed that phenylpropanoid phenolic acids and their derivatives are the core functional substances responsible for the antioxidant capacity and nutritional properties of white maize (Daji et al., 2023; Zhao et al., 2023).

The plant microbiome is a key determinant of host phenotypic plasticity, physiological status, reproductive capacity, and growth and development (Bulgarelli et al., 2013; Turner et al., 2013). Accordingly, the diversity and metabolic potential of endophytic microbial communities are increasingly acknowledged as critical factors modulating host biological traits (Compant et al., 2019; Trivedi et al., 2020). Plant-microbe interactions regulate intricate metabolic networks, through which symbiotic microorganisms enhance plant nutrient uptake, synthesize bioactive metabolites or phytohormones, and further reshape plant growth, development, and metabolite profiles. These microbial-mediated effects ultimately determine crop performance and grain quality (Fadiji & Babalola, 2020; Pang et al., 2021).

The present comparative metabolomics and microbiome analysis of yellow and white maize revealed significant differences in kernel bacterial and fungal community assembly between the two cultivars. Although fungal community richness showed no significant differences between the two cultivars, the fungal alpha diversity (Shannon index) was significantly higher in yellow maize (H) than in white maize (B). Moreover, partial least squares discriminant analysis (PLS-DA) clearly distinguished the bacterial and fungal communities of the two maize cultivars, demonstrating that host genotype and kernel metabolic profiles are the dominant drivers of endophytic microbial colonization (Peiffer et al., 2013; Walters et al., 2018). Genotype-dependent selection constructs unique microenvironmental niches that selectively filter and recruit specific microbial colonizers (Stringlis et al., 2018).

*Pseudomonas* exhibited remarkably high relative abundance in white maize kernels, and Chloroflexi emerged as a distinctively dominant phylum. *Pseudomonas* species possess highly versatile metabolic capabilities and are well-documented for their ability to modulate host stress responses and utilize complex plant carbohydrates (Santoyo et al., 2016). Such metabolic activity may provide additional carbon sources or alter metabolic precursors, potentially contributing to the distinct secondary metabolite profiles—including phenolic compounds—observed in white maize. Conversely, members of the order Rhizobiales and the genus *Tepidiamorphus* were highly abundant in yellow maize kernels. Previous studies have demonstrated that plant-associated Rhizobiales can significantly enhance host nutrient acquisition and upregulate specific secondary metabolite levels in host tissues (Garrido-Oter et al., 2018). Therefore, we hypothesize that these dominant bacteria in yellow maize may similarly influence isoprenoid metabolism and plastid development, and thereby contribute to the accumulation of zeaxanthin and lutein-related metabolites; this remains an association-based hypothesis that requires experimental validation.

Ascomycota is the predominant endophytic fungal phylum in cereal crops, and its structure is significantly correlated with grain antioxidant levels and nutritional composition, indicating that endophytic fungi actively modulate seed chemical properties (Hardoim et al., 2015). In this study, Ascomycota dominated the fungal communities of both maize cultivars. Further LEfSe analysis showed that two Ascomycota classes, Dothideomycetes and Sordariomycetes, were significantly enriched in yellow maize (H) kernels, suggesting that these specific fungal taxa influence the phytochemical composition of yellow maize.

LEfSe analysis also revealed distinct fungal enrichment patterns between the two cultivars: Aspergillus was highly aggregated in white maize (B) kernels, whereas Fusarium and Cladosporium were enriched in yellow maize (H). As common endophytic fungi and opportunistic pathogens in maize, Aspergillus and Fusarium colonization can trigger localized host defense responses (Bacon et al., 2001). Since phenolic acids, alkaloids, and other secondary metabolites serve as core defensive chemicals in cereal tissues (Pusztahelyi et al., 2015), microbial colonization may activate defense-related biosynthetic pathways, further shaping the unique metabolic and functional profiles of maize kernels.

It is important to emphasize that most of the endophytic taxa detected here are, by definition, also capable of behaving as plant pathogens, and several of them have direct implications for food safety and human health. Aspergillus dominated the white maize (B) kernels, and Aspergillus flavus and closely related species are the principal pre-harvest producers of aflatoxins, which rank among the most hepatocarcinogenic naturally occurring food contaminants (Klich, 2007). Fusarium, recovered here as a discriminant biomarker of yellow (H) kernels, is a well-characterized endophytic and pathogenic fungus of maize and a major producer of fumonisins and other mycotoxins that accumulate in kernels and pose chronic health risks to consumers (Bacon et al., 2008; Fisher et al., 2012). Among the bacterial genera detected in this study, Pseudomonas, Staphylococcus, and Corynebacterium include opportunistic human pathogens whose occurrence in an edible plant compartment warrants attention (Compant et al., 2010; Hallmann et al., 1997). DNA-based amplicon profiling cannot distinguish viable from non-viable cells, provides no information on toxin accumulation, and was not accompanied by mycotoxin quantification. Nevertheless, the consistent detection of these taxa in a food matrix indicates that endophyte-informed biofortification strategies must be coupled with routine mycotoxin and microbiological monitoring, and that the food-safety dimension of kernel endophytes deserves explicit evaluation in future work.

Metabolomic profiling and KEGG enrichment analyses demonstrated that pathways associated with phenylalanine, tyrosine, and tryptophan biosynthesis, as well as ABC transporter pathways, were significantly enriched in maize kernels. The aromatic amino acid biosynthesis pathway provides crucial precursors for numerous secondary metabolites, including monolignols, flavonoids, and alkaloids identified in our study (Tzin & Galili, 2010). Additionally, the widespread accumulation of monoterpenoids and lipid metabolites was consistent with the high abundance of lutein and zeaxanthin, which are synthesized via the carotenoid biosynthesis pathway (Wurtzel et al., 2012). These lipophilic compounds and photoprotective pigments are expected to contribute to the nutritional value of yellow maize through their interaction with human antioxidant systems. Furthermore, enriched ABC transporters facilitate the transmembrane transport and subcellular compartmentalization of diverse secondary metabolites, which is consistent with a role in kernel maturation and pigment formation (Hwang et al., 2016).

Notably, the plant hormone signal transduction pathway exhibited significant differential enrichment between the two maize cultivars. Plant hormones act as upstream signaling regulators that interact with multiple metabolic networks to coordinate plant secondary metabolism (Kaundal et al., 2017; Ku et al., 2018). Differential hormone signaling in yellow and white maize may regulate the expression of downstream transcription factors, thereby plausibly redirecting metabolic flux in a microbiome-associated manner (Eichmann et al., 2021). Collectively, this tripartite regulatory network consisting of host genetics, phytohormone signaling, and the endophytic microbiome plausibly underlies the divergent metabolic characteristics of the two maize cultivars. This network is consistent with the extensive carotenoid accumulation in yellow maize and the defense-related phenolic metabolite enrichment in white maize, ultimately establishing the distinct nutritional and microbial landscapes of yellow and white maize cultivars.

## 5 Conclusion

In summary, this study conducts a comprehensive comparative analysis of carotenoid accumulation, endophytic microbiome composition, and global metabolic profiles between yellow and white maize cultivars. Phenotypically, yellow maize exhibited substantially higher accumulations of lutein and zeaxanthin, two pivotal photoprotective pigments, whereas these compounds are detected only in trace quantities in white maize. Microbiome profiling reveals that bacterial alpha diversity remains comparable between the two cultivars, while yellow maize harbors significantly higher fungal diversity. The bacterial genera *Bdellovibrio*, *Neisseria*, *Acidaminobacter*, and *Syntrophobacter* and the fungal genera *Fusarium*, *Acremonium*, *Phoma*, and *Cladosporium* were enriched in yellow maize, whereas *Aspergillus* was enriched in white maize, whose bacterial community was dominated by *Pseudomonas*; *Tepidiamorphus* was a dominant genus in yellow maize but was not retained as a significant biomarker by LEfSe. Functional prediction analyses further demonstrate pronounced divergences in the ecological guilds of fungal communities associated with the two cultivars. Untargeted metabolomics confirms the distinct metabolic signatures of yellow and white maize, with a total of 313 significantly differentially annotated metabolites, primarily including lipids, organic acids, and phenylpropanoids. The observed metabolic differences are associated with several highly enriched pathways, such as phenylalanine, tyrosine, and tryptophan biosynthesis, plant hormone signal transduction, and ABC transporter pathways. Collectively, this multi-omics study advances our holistic understanding of the molecular and microbial mechanisms underlying maize grain quality formation. It also provides valuable microbial and metabolic targets for future precision maize breeding and nutritional biofortification practices.

Several limitations of this work should, however, be acknowledged. First, grain colour and cultivar genotype were not independently manipulated, so the observed differences cannot be attributed unambiguously to colour per se rather than to cultivar-specific genetic backgrounds. Second, because the design is observational, the detected microbe–metabolite associations do not demonstrate causal relationships, and statements about endophyte-mediated regulation of carotenoid or phenolic accumulation remain hypotheses rather than demonstrated mechanisms. Third, amplicon-based profiling resolves metabolite annotations at MSI Level 2, does not distinguish viable from non-viable microorganisms, and was not accompanied by mycotoxin quantification, although several detected taxa are known mycotoxin producers. Future work should therefore combine cultivation-independent and cultivation-based approaches with isotope tracing, gnotobiotic kernel systems, and targeted carotenoid and mycotoxin quantification to test whether the candidate endophytes identified here causally modulate maize grain nutritional quality.

## Authors contribution

Q.L., Y.Z. and S.Y. conceived and designed the study. Q.L. and H.L. developed the methodology and implemented the computational framework. Y.Z. and H.H. performed the investigation and curated the experimental data. K.L. carried out the formal analysis and validated the results. H.L. generated the figures and data visualizations. L.L. contributed resources and oversaw project administration. X.W. and S.Y. supervised the research, acquired funding, and coordinated the collaboration between the two institutions. Q.L. wrote the original draft of the manuscript, and all authors reviewed, revised, and approved the final version. X.W. and S.Y. are the corresponding authors and are responsible for the integrity of the work as a whole.

## CRediT authorship contribution statement

**Qingshan Li:** Writing – original draft, Data curation, Conceptualization. **Yu Zu:** Supervision, Software. **Hongda Liao:** Methodology, Investigation. **Haidong Huang:** Software, Resources, Methodology. **Kexin Lu:** Data curation. **Lan Lan:** Writing – original draft. **XiQin Wei:** Writing – review & editing, Funding acquisition. **Shangdong Yang:** Writing – review & editing, Conceptualization.

## Funding

Shangdong Yang received funding from The “Sharp Edge” Action Plan (a major special project in Guangxi) (Guike JF2504850035). Guangxi Science and Technology Base & Talent Special Project–Project of the Subtropical State Key Laboratory (Guike AD25069107). Guangxi University and Bama County Industry-University-Research Project (Barenke 20220005).

## Declaration of competing interest

The authors declare that they have no known competing financial interests or personal relationships that could have appeared to influence the work reported in this paper.

## Data Availability

The data that has been used is confidential.

## Appendix A Supplementary data

Supplementary Table S1 Overview Table of Metabolite Annotation Information

Supplementary Table S2 Key metabolites identified in the significant modules of WGCNA analysis for different corn varieties

## References

[bb0005] Awika J.M. (2011). Advances in cereal science: Implications to food processing and health promotion.

[bb0010] Babu R., Rojas N.P., Gao S., Yan J., Pixley K. (2013). Validation of the effects of molecular marker polymorphisms in LcyE and CrtRB1 on provitamin a concentrations for 26 tropical maize populations. Theoretical and Applied Genetics.

[bb0015] Bacon C.W., Glenn A.E., Yates I.E. (2008). Fusarium verticillioides: Managing the endophytic association with maize for reduced fumonisins accumulation. Toxin Reviews.

[bb0020] Bacon C.W., Yates I.E., Hinton D.M., Meredith F. (2001). Biological control of *fusarium moniliforme* in maize. Environmental Health Perspectives.

[bb0025] Bai L., Kim E.-H., DellaPenna D., Brutnell T.P. (2009). Novel lycopene epsilon cyclase activities in maize revealed through perturbation of carotenoid biosynthesis. The Plant Journal.

[bb0035] Blaženović I., Kind T., Ji J., Fiehn O. (2018). Software tools and approaches for compound identification of LC-MS/MS data in metabolomics. Metabolites.

[bb0040] Bulgarelli, D., Schlaeppi, K., Spaepen, S., Ver Loren van Themaat, E., & Schulze-Lefert, P. (2013). Structure and functions of the bacterial microbiota of plants. Annual Review of Plant Biology, 64, 807–838. Doi: 10.1146/annurev-arplant-050312-120106. 23373698

[bb0045] Compant S., Clément C., Sessitsch A. (2010). Plant growth-promoting bacteria in the rhizo- and endosphere of plants: Their role, colonization, mechanisms involved and prospects for utilization. Soil Biology and Biochemistry.

[bb0050] Compant S., Samad A., Faist H., Sessitsch A. (2019). A review on the plant microbiome: Ecology, functions, and emerging trends in microbial application. Journal of Advanced Research.

[bb0055] Daji O.G., Green E., Adebo O.A. (2023). Nutritional and phytochemical composition of mahewu (a southern African fermented food product) derived from white and yellow maize (*Zea mays* L.). Fermentation.

[bb0060] Diepenbrock C.H., Ilut D.C., Magallanes-Lundback M., Kandianis C.B., Lipka A.E., Bradbury P.J., DellaPenna D. (2021). Eleven biosynthetic genes explain the majority of natural variation in carotenoid levels in maize grain. The Plant Cell.

[bb0065] Eggersdorfer M., Wyss A. (2018). Carotenoids in human nutrition and health. Archives of Biochemistry and Biophysics.

[bb0070] Eichmann R., Richards L., Schäfer P. (2021). Hormones as go-betweens in plant microbiome assembly. The Plant Journal.

[bb0075] Fadiji A.E., Babalola O.O. (2020). Elucidating mechanisms of endophytes used in plant protection and other bioactivities with multifunctional prospects. Frontiers in Bioengineering and Biotechnology.

[bb0080] Fiehn O. (2002). Metabolomics—The link between genotypes and phenotypes. Plant Molecular Biology.

[bb0085] Fisher M.C., Henk D.A., Briggs C.J., Brownstein J.S., Madoff L.C., McCraw S.L., Gurr S.J. (2012). Emerging fungal threats to animal, plant and ecosystem health. Nature.

[bb0090] Gardes M., Bruns T.D. (1993). ITS primers with enhanced specificity for basidiomycetes—Application to the identification of mycorrhizae and rusts. Molecular Ecology.

[bb0095] Garrido-Oter R., Nakano R.T., Dombrowski N., Ma K.-W., McHardy A.C., Schulze-Lefert P. (2018). Modular traits of the *Rhizobiales* root microbiota and their evolutionary relationship with symbiotic rhizobia. Cell Host & Microbe.

[bb0100] Gowda M., Das B., Makumbi D., Babu R., Semagn K., Mahuku G., Prasanna B.M. (2015). Genome-wide association and genomic prediction of resistance to maize lethal necrosis disease in tropical maize germplasm. Theoretical and Applied Genetics.

[bb0105] Gwirtz J.A., Garcia-Casal M.N. (2014). Processing maize flour and corn meal food products. Annals of the New York Academy of Sciences.

[bb0110] Hallmann J., Quadt-Hallmann A., Mahaffee W.F., Kloepper J.W. (1997). Bacterial endophytes in agricultural crops. Canadian Journal of Microbiology.

[bb0115] Hardoim P.R., van Overbeek L.S., Berg G., Pirttilä A.M., Compant S., Campisano A., Sessitsch A. (2015). The hidden world within plants: Ecological and evolutionary considerations for defining functioning of microbial endophytes. Microbiology and Molecular Biology Reviews.

[bb0120] Harjes C.E., Rocheford T.R., Bai L., Brutnell T.P., Kandianis C.B., Sowinski S.G., Buckler E.S. (2008). Natural genetic variation in lycopene epsilon cyclase tapped for maize biofortification. Science.

[bb0125] Hwang J.-U., Song W.-Y., Hong D., Ko D., Yamaoka Y., Jang S., Yim S., Lee E., Khare D., Kim K., Palmgren M., Yoon H., Martinoia E., Lee Y. (2016). Plant ABC transporters enable many unique aspects of a terrestrial plant's lifestyle. Molecular Plant.

[bb0130] Kaundal A., Ramu V.S., Oh S., Lee S., Pant B., Lee H.-K., Mysore K.S. (2017). General control nonrepressible4 degrades 14-3-3 and the RIN4 complex to regulate stomatal aperture with implications on nonhost resistance and drought tolerance. The Plant Cell.

[bb0135] Kgoale S.D., Jimoh A.A., Tugizimana F., Abrahams A., Adebo O.A. (2026). Targeted metabolomics to quantitatively profile changes in amino acids and phenolics at every step of amahewu production from two *Zea mays* L. cultivars. Frontiers in Nutrition.

[bb0140] Klich M.A. (2007). Aspergillus flavus: The major producer of aflatoxin. Molecular Plant Pathology.

[bb0145] Klindworth A., Pruesse E., Schweer T., Peplies J., Quast C., Horn M., Glöckner F.O. (2013). Evaluation of general 16S ribosomal RNA gene PCR primers for classical and next-generation sequencing-based diversity studies. Nucleic Acids Research.

[bb0150] Krinsky N.I., Johnson E.J. (2005). Carotenoid actions and their relation to health and disease. Molecular Aspects of Medicine.

[bb0155] Ku Y.-S., Sintaha M., Cheung M.-Y., Lam H.-M. (2018). Plant hormone signaling crosstalks between biotic and abiotic stress responses. International Journal of Molecular Sciences.

[bb0165] Luo N., Meng Q., Feng P., Qu Z., Yu Y., Liu D.L., Wang P. (2023). China can be self-sufficient in maize production by 2030 with optimal crop management. Nature Communications.

[bb0170] Muzhingi T., Yeum K.-J., Russell R.M., Johnson E.J., Qin J., Tang G. (2008). Determination of carotenoids in yellow maize, the effects of saponification and food preparations. International Journal for Vitamin and Nutrition Research.

[bb0175] Nuss E.T., Tanumihardjo S.A. (2010). Maize: A paramount staple crop in the context of global nutrition. Comprehensive Reviews in Food Science and Food Safety.

[bb0180] Ortiz D., Rocheford T., Ferruzzi M.G. (2016). Influence of temperature and humidity on the stability of carotenoids in biofortified maize (Zea mays L.) genotypes during controlled postharvest storage. Journal of Agricultural and Food Chemistry.

[bb0185] Owens B.F., Lipka A.E., Magallanes-Lundback M., Tiede T., Diepenbrock C.H., Kandianis C.B., Rocheford T. (2014). A foundation for provitamin a biofortification of maize: Genome-wide association and genomic prediction models of carotenoid levels. Genetics.

[bb0190] Pang Z., Chen J., Wang T., Gao C., Li Z., Guo L., Xu J., Cheng Y. (2021). Linking plant secondary metabolites and plant microbiomes: A review. Frontiers in Plant Science.

[bb0195] Peiffer J.A., Spor A., Koren O., Jin Z., Tringe S.G., Dangl J.L., Ley R.E. (2013). Diversity and heritability of the maize rhizosphere microbiome under field conditions. Proceedings of the National Academy of Sciences.

[bb0200] Pérez-Massot E., Banakar R., Gómez-Galera S., Zorrilla-López U., Sanahuja G., Arjó G., Zhu C. (2013). The contribution of transgenic plants to better health through improved nutrition: Opportunities and constraints. Genes & Nutrition.

[bb0205] Pixley K., Palacios-Rojas N., Babu R., Mutale R., Surles R., Simpungwe E., Tanumihardjo S.A. (2013). Carotenoids and human health.

[bb0210] Pusztahelyi T., Holb I.J., Pócsi I. (2015). Secondary metabolites in fungus-plant interactions. Frontiers in Plant Science.

[bb0215] Ranum P., Peña-Rosas J.P., Garcia-Casal M.N. (2014). Global maize production, utilization, and consumption. Annals of the New York Academy of Sciences.

[bb0220] Santoyo G., Moreno-Hagelsieb G., Orozco-Mosqueda M.d.C., Glick B.R. (2016). Plant growth-promoting bacterial endophytes. Microbiological Research.

[bb0225] Schulz B., Boyle C. (2005). The endophytic continuum. Mycological Research.

[bb0230] Stringlis I.A., Yu K., Feussner K., de Jonge R., Van Bentum S., Van Verk M.C., Pieterse C.M.J. (2018). MYB72-dependent coumarin exudation shapes root microbiome assembly to promote plant health. Proceedings of the National Academy of Sciences.

[bb0235] Sumner L.W., Amberg A., Barrett D., Beale M.H., Beger R., Daykin C.A., Viant M.R. (2007). Proposed minimum reporting standards for chemical analysis. Metabolomics.

[bb0240] Sun, T., Rao, S., Zhou, X., & Li, L. (2022). Plant carotenoids: Recent advances and future perspectives. Molecular Horticulture, 2, 2. Doi: 10.1186/s43897-022-00023-2. PMC10515021 37789426

[bb0245] Suwarno W.B., Pixley K.V., Palacios-Rojas N., Kaeppler S.M., Babu R. (2014). Formation of heterotic groups and understanding genetic effects in a provitamin a biofortified maize breeding program. Crop Science.

[bb0250] Trivedi P., Leach J.E., Tringe S.G., Sa T., Singh B.K. (2020). Plant–microbiome interactions: From community assembly to plant health. Nature Reviews Microbiology.

[bb0255] Turner T.R., James E.K., Poole P.S. (2013). The plant microbiome. Genome Biology.

[bb0260] Tzin, V., & Galili, G. (2010). The biosynthetic pathways for shikimate and aromatic amino acids in Arabidopsis thaliana. The Arabidopsis Book, 8, e0132. Doi: 10.1199/tab.0132. PMC3244902 22303258

[bb0265] Walters W.A., Jin Z., Youngblut N., Wallace J.G., Sutter J., Zhang W., Ley R.E. (2018). Large-scale replicated field study of maize rhizosphere identifies heritable microbes. Proceedings of the National Academy of Sciences.

[bb0270] Wurtzel E.T., Cuttriss A., Vallabhaneni R. (2012). Maize provitamin a carotenoids, current resources, and future metabolic engineering challenges. Frontiers in Plant Science.

[bb0275] Yan J., Kandianis C.B., Harjes C.E., Bai L., Kim E.-H., Yang X., Rocheford T. (2010). Rare genetic variation at *Zea mays* crtRB1 increases β-carotene in maize grain. Nature Genetics.

[bb0280] Zhao H., Qin Y., Xiao Z., Sun Q., Gong D., Qiu F. (2023). Revealing the process of storage protein rebalancing in high quality protein maize by proteomic and transcriptomic analyses. Journal of Integrative Agriculture.

[bb0285] Žilić S., Serpen A., Akıllıoğlu G., Gökmen V., Vančetović J. (2012). Phenolic compounds, carotenoids, anthocyanins, and antioxidant capacity of colored maize (Zea mays L.) kernels. Journal of Agricultural and Food Chemistry.

